# Enhancing durability of CIS43 monoclonal antibody by Fc mutation or AAV delivery for malaria prevention

**DOI:** 10.1172/jci.insight.143958

**Published:** 2021-02-08

**Authors:** Neville K. Kisalu, Lais D. Pereira, Keenan Ernste, Yevel Flores-Garcia, Azza H. Idris, Mangaiarkarasi Asokan, Marlon Dillon, Scott MacDonald, Wei Shi, Xuejun Chen, Amarendra Pegu, Arne Schön, Fidel Zavala, Alejandro B. Balazs, Joseph R. Francica, Robert A. Seder

**Affiliations:** 1 Vaccine Research Center (VRC), National Institute of Allergy and Infectious Diseases (NIAID), NIH, Bethesda, Maryland, USA.; 2 Malaria Research Institute, Johns Hopkins Bloomberg School of Public Health, Baltimore, Maryland, USA.; 3 Ragon Institute of Massachusetts General Hospital, Massachusetts Institute of Technology and Harvard University, Cambridge, Massachusetts, USA.; 4 Department of Biology, Johns Hopkins University, Baltimore, Maryland, USA.

**Keywords:** Infectious disease, Immunoglobulins, Malaria, Skin

## Abstract

CIS43 is a potent neutralizing human mAb that targets a highly conserved “junctional” epitope in the *Plasmodium falciparum* (Pf) circumsporozoite protein (PfCSP). Enhancing the durability of CIS43 in vivo will be important for clinical translation. Here, 2 approaches were used to improve the durability of CIS43 in vivo while maintaining potent neutralization. First, the Fc domain was modified with the LS mutations (CIS43LS) to increase CIS43 binding affinity for the neonatal Fc receptor (FcRn). CIS43LS and CIS43 showed comparable in vivo protective efficacy. CIS43LS had 9- to 13-fold increased binding affinity for human (6.2 nM versus 54.2 nM) and rhesus (25.1 nM versus 325.8 nM) FcRn at endosomal pH 6.0 compared with CIS43. Importantly, the half-life of CIS43LS in rhesus macaques increased from 22 days to 39 days compared with CIS43. The second approach for sustaining antibody levels of CIS43 in vivo is through adeno-associated virus (AAV) expression. Mice administered once with AAV-expressing CIS43 had sustained antibody levels of approximately 300 μg/mL and mediated protection against sequential malaria challenges up to 36 weeks. Based on these data, CIS43LS has advanced to phase I clinical trials, and AAV delivery provides a potential next-generation approach for malaria prevention.

## Introduction

Malaria is a mosquito-borne parasitic disease causing high morbidity and mortality primarily in infants and young children in sub-Saharan Africa. Several interventions have significantly contributed to the decrease of malaria case incidence and mortality, including vector control, insecticide-treated bednets, and seasonal malaria chemoprevention. However, reductions in malaria cases have plateaued globally since 2015 and are actually increasing in some countries (1, 2). Thus, there is an urgent need to develop new approaches for controlling and eventually eradicating malaria.

The most transformative modality to control malaria would be a vaccine that provides high-level and durable protection. RTS,S, a truncated version of *Plasmodium falciparum* (Pf) circumsporozoite protein (PfCSP) containing NANP repeats and the C- terminal region administered with the AS01 adjuvant, induces approximately 50% protection at 1 year and approximately 30% protection after 4 years, primarily due to the high level of antibodies required for protection (3, 4). While some vaccine strategies focused on T cell–mediated protection such as whole sporozoite–based (SPZ-based) vaccines conferred high-level, durable protection of approximately 1 year in malaria-naive individuals following controlled human malaria infection (CHMI; refs. 5–7), there is more limited immunity and protection in malaria-endemic regions against CHMI (8) or in areas of intense natural transmission (9). Factors that may influence vaccine efficacy include prior malaria exposure (9, 10), parasite diversity (11, 12), and age (13, 14). Thus, an alternative immune approach independent of the potential factors that limit immunity by vaccination — one that would induce high-level protection for defined periods of time — is through passive immunization with a highly potent mAb.

We recently reported on the discovery of CIS43, a human mAb isolated from a subject immunized with an attenuated Pf whole SPZ vaccine (Sanaria PfSPZ Vaccine; ref. 15) that was protected against CHMI (16). CIS43 preferentially recognizes the “junctional” epitope positioned between the N-terminus and the central repeat domain of PfCSP, which is highly conserved across 99% of Pf strains. Passive transfer of CIS43 provided high-level, sterile protection in 2 different mouse models of malaria infection (17). In addition to potency, the clinical utility of mAbs such as CIS43 will be strongly influenced by improving the antibody half-life (*t_1/2_*).

Here, 2 distinct strategies are used to extend the durability of CIS43 in vivo. First, we introduced the LS mutations (leucine [L] and serine [S] amino acids) in the Fc (constant) region of CIS43 to enhance its binding affinity for the neonatal Fc receptor (FcRn), to limit degradation of the antibody and increase recycling (18, 19). Second, vectored immunoprophylaxis (VIP), an adeno-associated virus (AAV) gene-based delivery strategy that has been used for expressing antibodies in vivo against a number of viral antigens or PfCSP, was used to express CIS43 (20–24). Together, the data presented provide evidence for optimizing the ability of a malaria mAb to mediate durable protection in vivo.

## Results

### Generation of CIS43LS mAb.

To improve the durability of CIS43, this mAb was modified by site-directed mutagenesis to introduce the LS point mutations, which replaced methionine with leucine at amino acid position 451 and asparagine with serine at amino acid position 457 in the CH3 domain of the Fc region (M451L/N457S) (Supplemental Figure 1; supplemental material available online with this article; https://doi.org/10.1172/jci.insight.143958DS1). The LS mutations increase the binding affinity of the Fc region for FcRn, preventing its degradation and thereby increasing the serum *t_1/2_* (18, 19).

**Figure 1:**
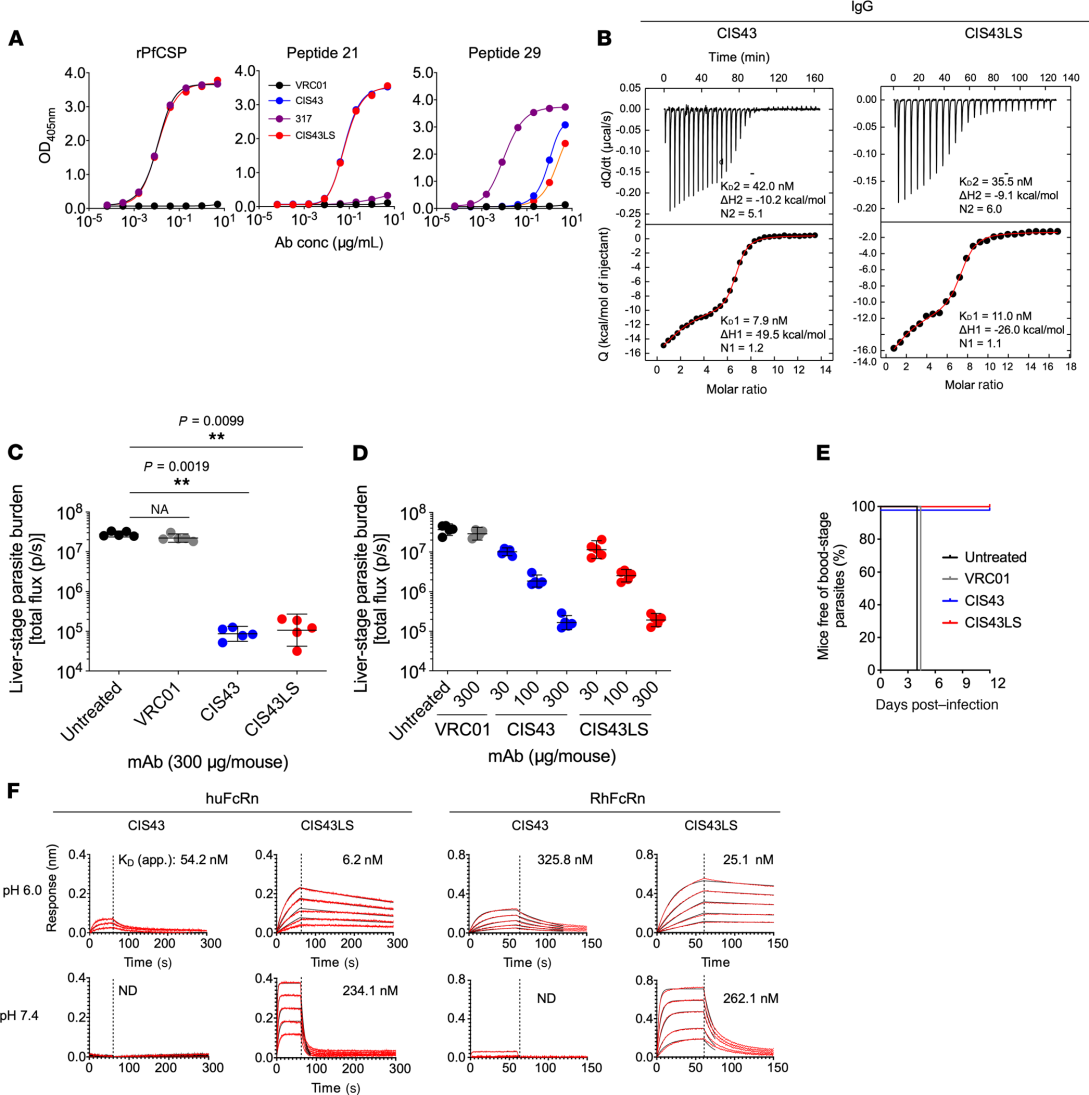
Characterization of CIS43LS. (**A**) Binding of CIS43LS to rPfCSP and PfCSP peptides by ELISA. CIS43, VRC01 (human anti-HIV-1 IgG1), and 317 (human antibody specific for the NANP-repeat region of PfCSP) were used as control antibodies. (**B**) Thermodynamic parameters and stoichiometry of binding of CIS43LS to rPfCSP by isothermal calorimetry. The *K_D_*, change in Gibbs energy (ΔG) of binding, enthalpy (ΔH), entropy contribution to Gibbs energy (−TΔS), and stoichiometry (N) are shown. Data displayed are representative of 2 independent experiments. (**C**) Protective effect of CIS43LS on liver burden. Following passive transfer of CIS43LS or CIS43, C57BL/6 mice were challenged i.v. with Pb-PfCSP-LUC SPZ before imaging by IVIS. Differences in liver-stage parasite burden reduction between the VRC01, CIS43, or CIS43LS group compared with the untreated group were determined using the nonparametric Kruskal-Wallis test for multiple comparisons with Dunn’s correction. *P* values are displayed on the panels. **P = 0.0019 (CIS43) or 0.0099 (CIS43LS). (**D**) Protective effect on liver burden by CIS43LS at varying concentrations (30–300 μg/mL). Data represent geometric mean with 95% CI (**C** and **D**). (**E**) Sterile protection by CIS43LS following infection by mosquito bites. C57BL/6 mice were challenged with infected mosquitoes following passive transfer of CIS43LS or CIS43 (300 μg per mouse). The presence of parasites was determined through Giemsa staining of blood up to 12 days following challenge. Kaplan-Meier curves, analyzed by the log rank test, show frequencies of mice free of parasites as determined by Giemsa staining of blood. Differences between CIS43LS and CIS43 as compared with untreated mice are shown (*P =* 0.0001). *n =* 5 per group (**C**–**E**). (**F**) Apparent affinity of CIS43LS to human and rhesus FcRn at acidic pH 6.0 and physiological pH 7.4. Antibody binding curves are shown in red (raw data) and black (fitted data). The apparent affinity is displayed as *K_D_* in nM. A representative of 2 independent experiments is shown. ND, no fit could be determined.

To establish that CIS43LS retained the biophysical properties of CIS43, the binding specificity, affinity, and stoichiometry were characterized. CIS43LS showed dose-dependent binding to recombinant PfCSP (rPfCSP) by ELISA similar to CIS43 with an effective concentration (EC_50_) of 0.039 and 0.037 μg/mL, respectively (Figure 1A). mAb 317, a human antibody specific for the NANP-repeat region of PfCSP used as a control antibody, bound to rPfCSP with an EC_50_ of 0.011 μg/mL (Figure 1A). Epitope mapping of CIS43LS and CIS43 confirmed comparable specificity with potent binding to peptide 21, the preferred junctional epitope of CIS43, with an EC_50_ of 0.06 μg/mL and binding to a NANP-containing peptide (peptide 29) with an EC_50_ of 5.1 μg/mL. mAb 317 did not bind to peptide 21 and had an EC_50_ of 0.010 for binding to peptide 29 (Figure 1A). Similar to CIS43, the stoichiometry and thermodynamic parameters of CIS43LS measured by isothermal titration calorimetry (ITC) showed 2 sequential binding events to rPfCSP (Figure 1B). The first binding event to the junctional epitope involved a single binding site per antibody with an apparent affinity of 11.0 nM (CIS43LS) and 7.9 nM (CIS43), whereas the second binding event encompassed 5–6 additional sites within the NANP repeat region with an apparent affinity of 35.5 nM for CIS43LS and 42.0 nM for CIS43 (Figure 1B). These data show that the LS mutations did not disrupt the binding of CIS43 to its cognate antigen and confirmed that the junctional epitope is the preferred epitope for this mAb.

### CIS43LS mediates protection in vivo in mice.

To determine whether CIS43LS can protect against malaria challenge in vivo and to compare its potency with CIS43, mice were challenged with a transgenic parasite strain of chimeric *Plasmodium berghei*–expressing (Pb-expressing) PfCSP and luciferase (Pb-PfCSP-LUC; ref. 25) following passive transfer of the mAb. First, in 2 independent experiments, we assessed the ability of CIS43 and CIS43LS to mediate reduction of liver-stage parasite burden as measured by bioluminescence in vivo in C57BL/6 mice following challenge with 2000 Pb-PfCSP-LUC SPZs (Pb-PfCSP-LUC SPZ) by i.v. injection (17). Passive administration of CIS43 or CIS43LS led to similar and significant reduction (~2.5 logs) of the liver-stage parasite burden in a dose-dependent manner (*P =* 0.0019 for CIS43 and *P =* 0.0099 for CIS43-LS; Figure 1, C and D) compared with untreated mice or compared with mice that received VRC01, a human anti–HIV-1 isotype control mAb. The protective capacity of CIS43LS was next assessed following challenge with infectious mosquito bites, the natural route of transmission for malaria. In 2 independent experiments, all mice that received 300 μg CIS43 or CIS43LS had no detectible parasites in blood (parasitemia) up to 12 days following infection (*P =* 0.0001, log-rank test) while the untreated or isotype control–treated mice were all parasitemic by day 4 (Figure 1E). Together, these data show that CIS43LS had comparable protective efficacy as CIS43 in vivo.

### CIS43LS shows pH-dependent increased affinity for FcRn.

FcRn binding increases antibody *t_1/2_* by preventing its degradation in lysosomes, allowing it to recycle back into circulation (18, 19). This association is pH dependent, whereby high binding occurs at low pH but not at physiological pH. To confirm that CIS43LS had this pH dependency, we measured the capacity of CIS43LS to bind to human and rhesus FcRn at endosomal or physiological pH (6.0 or 7.4, respectively) using biolayer interferometry. Indeed, at pH 6.0, CIS43LS had a higher apparent binding affinity to human (6.2 nM) and rhesus (25.1 nM) FcRn, compared with CIS43 binding to human (54.2 nM) and rhesus (325.8 nM) FcRn (Figure 1F and Supplemental Table 1). At pH 7.4, the affinity of CIS43LS for both humans (234.1 nM) and rhesus (262.1 nM) FcRn was substantially reduced, while there was limited to no detectable binding to either human or rhesus FcRn for CIS43 (Figure 1F and Supplemental Table 1).

To extend the analysis, the binding affinities of CIS43 and CIS43LS for all human Fcγ receptors (Supplemental Table 2) were assessed at physiologic pH 7.4. There were no significant differences in the binding between these 2 antibodies for any of the Fcγ receptors. Thus, the LS mutation primarily affected pH-dependent FcRn binding, the primary mechanism by which antibody *t_1/2_* is increased (18, 19, 26).

### Pharmacokinetics of CIS43LS in nonhuman primates (NHP).

**Figure 2:**
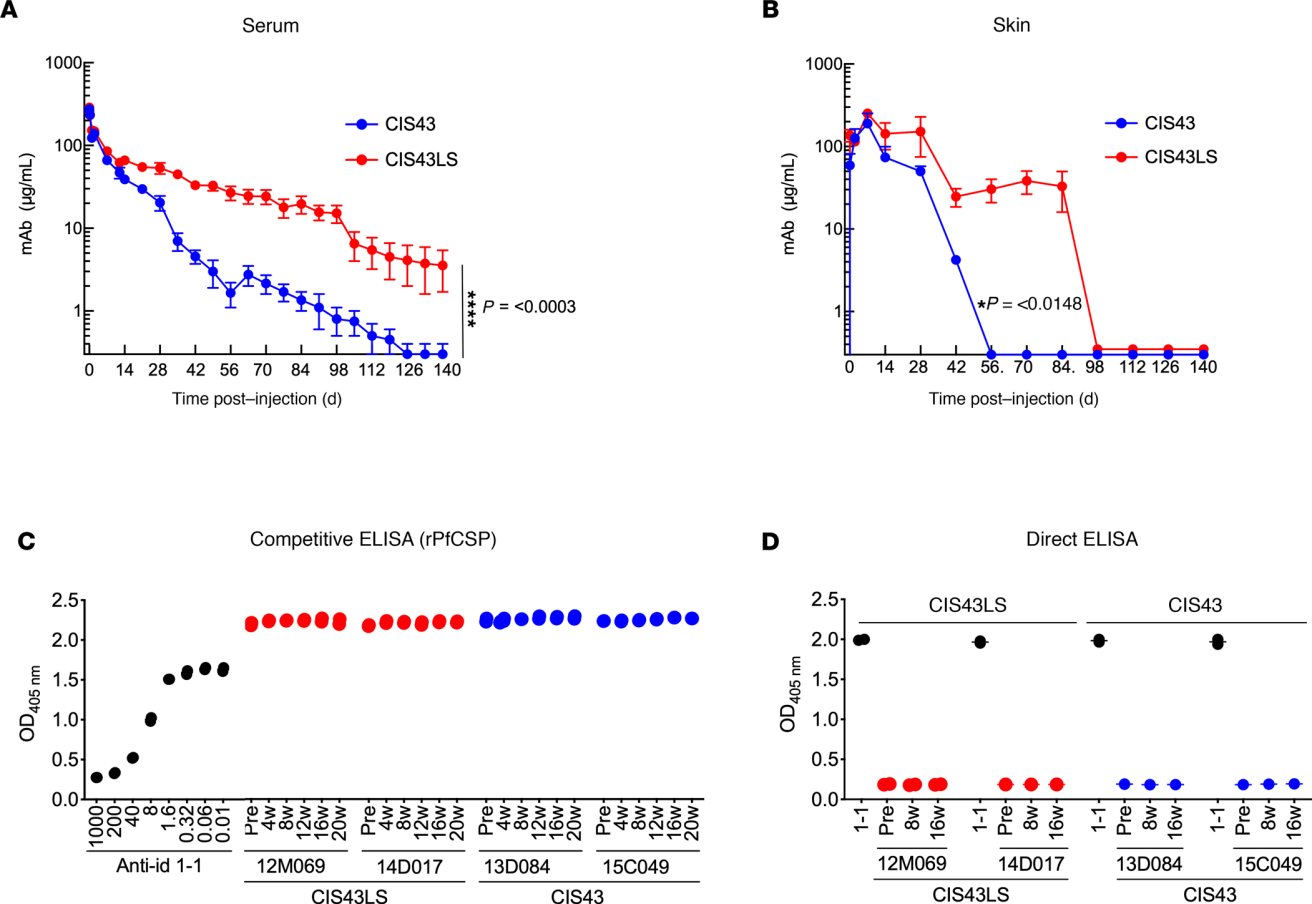
Pharmacokinetic study in Indian rhesus macaques. (**A**) Serum concentrations of CIS43LS were measured by ELISA following i.v. injection of 10 mg per kg body weight of CIS43 or CIS43LS in rhesus macaques. ****P = <0.0003. (**B**) The amount of mAb in skin punch homogenates from each group of CIS43- or CIS43LS-treated macaques displayed in **A** was quantitated over time and is shown. *P = <0.0148. Data represent the mean ± SD, and differences in mAb concentrations between both groups were determined using 2-way ANOVA (**A** and **B**). (**C** and **D**) ELISA to assess the NHP serum antibodies elicited against CIS43 or CIS43LS (antidrug antibodies, ADA). Competitive ADA ELISA (**C**) showing CIS43 or CIS43LS binding to rPfCSP in the presence of NHP sera before (Pre) or at the indicated time points after mAb infusion. mAb groups and animals infused with CIS43LS (12M069 and 14D017) or CIS43 (13D084 and 15C049) are indicated. Varying concentrations of mouse mAb 1-1, a CIS43 mouse anti-idiotype antibody, were used as a positive control for the competitive binding of CIS43LS or CIS43 to rPfCSP. Pre, preimmune serum collected at baseline prior to antibody infusion. OD_405nm_, optical density at 405 nm. Data collected at different time points from the animals that received CIS43LS (red) or CIS43 (blue) are shown; black, mAb 1-1. Direct ADA ELISA (**D**). CIS43LS or CIS43 was coated onto ELISA plates, and NHP sera were analyzed. Color codes are as in **C**. Data represent the mean ± SEM (**C** and **D**). For all panels, *n =* 2 per group.

The next studies focused on whether CIS43LS had enhanced *t_1/2_* compared with CIS43 in vivo. Accordingly, NHP can be a useful preclinical model for pharmacokinetic (PK) analysis of human mAbs based on high FcRn conservation compared with humans (27). Rhesus macaques (*Macaca mulatta*) were infused i.v. with 10 mg/kg of CIS43LS or CIS43, and the concentrations of both mAbs were assessed in serum and skin up to 140 days after infusion. The mean maximum serum antibody concentration (CMAX) was comparable between both mAbs (287.3 μg/mL for CIS43LS and 280.4 μg/mL for CIS43) and was attained within 1 hour after mAb administration (Supplemental Table 3). Although mAb titers were similar between CIS43 and CIS43LS up to 14 days after infusion (Figure 2A), CIS43LS exhibited a longer *t_1/2_* after this time point, resulting in a titer of approximately 15 μg/mL at 98 days compared with approximately 1 μg/mL for CIS43. Remarkably, CIS43LS remained detectable in serum for more than 140 days and persisted at significantly higher levels (~3.7 μg/mL) than CIS43, which was not detectible after 126 days following mAb administration (Figure 2A, P *<* 0.0003). We next analyzed the *t_1/2_* for each mAb using a 2-compartment model (Supplemental Table 3), and CIS43LS had an approximately 2.0-fold increase in *t_1/2_* (CIS43LS, 38.7 ± 7.8 days versus CIS43, 22.3 ± 2.2). The clearance of antibody, a measurement of plasma volume from which the antibody is entirely removed per day per Kg body weight, was decreased approximately 2.5-fold for CIS43LS compared with the parental antibody (CIS43, 5.8 ± 0.5 mL/day/kg; CIS43LS, 2.1 ± 0.3 mL/day/kg; Supplemental Table 3). Consequently, the AUC, which measures the exposure of all the body to the drug, for CIS43LS was ~3-fold larger than that for CIS43. These data are consistent with previous reports demonstrating prolonged mAb *t_1/2_* for FcRn binding mutants (18, 28, 29). As the skin has a critical role in the initiation of malaria infection, we also assessed whether the LS mutations would alter the distribution of CIS43 in this tissue. Skin biopsy samples were collected at various time points from the macaques that received CIS43 or CIS43LS, and antibody concentrations were measured in skin homogenates up to 140 days after infusion. Following antibody administration, each mAb showed a consistent titer pattern compared with that observed in the blood samples, with CIS43LS persisting longer in the skin (Figure 2B, P *=* 0.0148). Both mAb titers were similar for up to 7 days, but by day 14, CIS43 and CIS43LS titers were approximately 29.4 and 56.9 μg/mL, respectively. CIS43LS was still detectible in skin tissue for up to 84 days. By contrast, CIS43 titers became undetectable by 56 days after infusion. These data show that, as in blood, the LS mutant significantly increased the *t_1/2_* of CIS43 in the skin.

### CIS43LS does not induce anti-idiotypic responses in NHP.

Antidrug antibodies (ADA) may develop in response to passively administered mAbs, which may influence their neutralization capacity and durability (30, 31). To assess whether the LS mutant induced immune responses in passively immunized NHP, ADA was assessed up to 20 weeks after infusion. Two assays were used for ADA. First, a competition assay was performed in which CIS43LS or CIS43 was preincubated with serum before and after mAb administration; the mixture was then added to PfCSP-coated plates to determine any change of the mAb binding to PfCSP. As a positive control, a CIS43 anti-idiotype mAb was used to block CIS43LS or CIS43 binding to rPfCSP (Figure 2C). Both pre- and post-mAb infusion sera showed similar binding to PfCSP for all time points tested (Figure 2C), showing that there was no detectable ADA. A second direct approach for measuring ADA was used, in which plates were precoated with CIS43LS or CIS43, and sera were added and then detected with an anti–monkey IgG–specific secondary antibody. In this assay, no ADA could be detected at any time point (Figure 2D). Together, these data suggest that CIS43 and CIS43LS do not develop ADA in NHP up to 20 weeks after a single infusion.

### CIS43LS is not polyreactive.

A final consideration for clinical development of an antibody is to demonstrate the absence of polyreactivity or autoreactivity. CIS43LS was tested for binding to a panel of mammalian autoantigens and showed no evidence of autoreactivity for CIS43LS or CIS43 (Supplemental Figure 2, A and B).

### VIP for CIS43 using an AAV vector.

To provide an orthogonal approach for increasing antibody durability in vivo, VIP was used as a gene-based delivery approach (20–24). A previous study using an AAV vector–encoding 2A10, a mouse mAb against PfCSP, demonstrated high expression levels (>1000 μg/mL) but only partial protection following malaria challenge in mice after 8 weeks (23). Based on the enhanced potency of CIS43 compared with 2A10, we hypothesized that VIP with CIS43 would elicit higher protection than 2A10 for longer periods of time.

**Figure 3:**
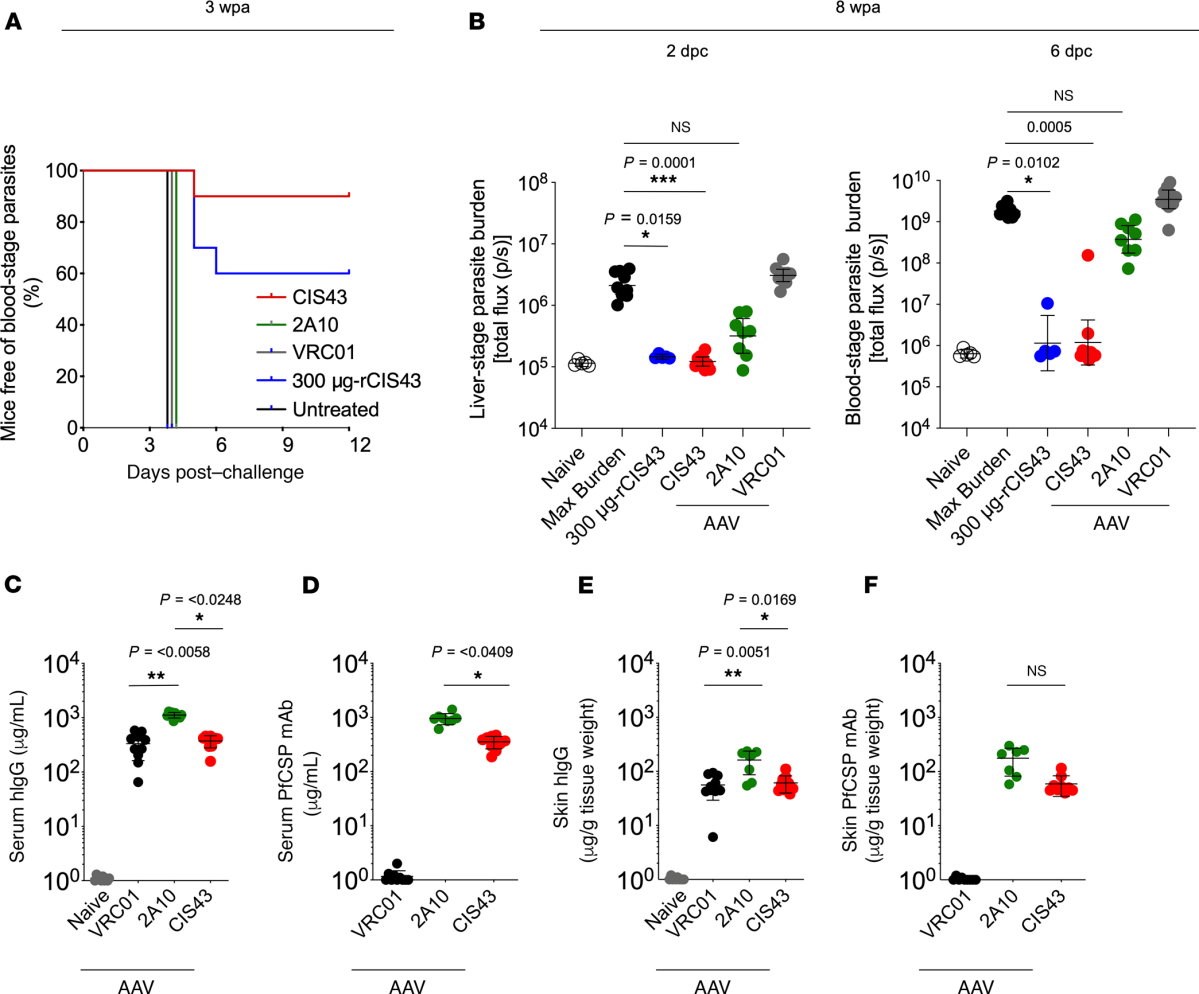
Protective efficacy and expression of PfCSP-AAV–induced mAbs in C57BL/6 mice. (**A**) Sterile protection by mosquito bite 3 weeks following intramuscular administration of 1 × 10^11^ genome copies (GC) AAV encoding CIS43, 2A10, or VRC01. C57BL/6 mice were challenged by mosquito bites 3 weeks following AAV administration of the indicated mAb. As a control, 300 μg-rCIS43 was administered 2 hours prior to challenge. Kaplan-Meier curves, analyzed using the log-rank test, show the frequencies of mice free of parasites. Differences between CIS43-AAV–administered or 300 μg rCIS43–treated mice and untreated mice are shown (*P =* 0.0001). wpa, weeks post-AAV administration; 300 μg-rCIS43, mice that passively received the protective dose of 300 μg of CIS43 at the time of challenge. *n =* 10 per group; 2A10-AAV, *n =* 8. (**B**) Protective effect of PfCSP-AAV expressed mAbs on liver burden 8 weeks following AAV administration. AAV-treated C57BL/6 albino mice were challenged i.v. with Pb-PfCSP-LUC SPZ and imaged by IVIS. Data represent the geometric mean with 95% CI. For liver burden (2 dpc), *P = 0.0159 and ***P = 0.0001; for parasitemia (6 dpc), *P = 0.0102 and ***P = 0.0005. Max burden, naive infected mice; 8 wpa, 8 weeks post-AAV administration; dpc, days postchallenge; hIgG; human IgG1. CIS43 and VRC01, *n =* 10 per group; 2A10, *n =* 8. (**C**–**F**) Serum hIgG (**C**) and PfCSP-specific mAb (**D**) levels, and skin hIgG (**E**) and PfCSP-specific mAb (**F**) levels from mice shown in **B** were measured by ELISA 8 wpa. Differences in antibody concentration, parasite liver burden, or parasitemia between each group and the untreated group were determined using the Kruskal-Wallis test for multiple comparisons with Dunn’s correction. *P* values are shown on panels. Means ± SD are displayed (**C**–**F**).

C57BL/6 mice were given a single intramuscular administration of 1 × 10^11^ genomic copies (GC) of the AAV-expressing CIS43, 2A10, or VRC01 (negative control) mAb. Three weeks after administration, the protective efficacy of AAV-encoded PfCSP mAbs against malaria infection was assessed in C57BL/6 mice challenged with infectious mosquito bites, providing evidence that the antibodies generated in vivo to PfCSP can inhibit SPZs in the skin (32, 33). Nine of 10 mice injected with CIS43-AAV and 6 of 10 mice that passively received 300 μg of CIS43 mAb as a positive control had no detectable parasitemia up to 12 days after infection (*P =* 0.0001, log-rank test). In contrast, all mice that received either 2A10-AAV or VRC01-AAV, or untreated mice, exhibited parasitemia by day 4 (Figure 3A). To extend these findings and assess durability of protection, C57BL/6 albino mice were challenged by the i.v. route 8 weeks after AAV administration with Pb-PfCSP-LUC SPZ to demonstrate that the in vivo–expressed antibodies can neutralize SPZs in the blood. CIS43-AAV–treated (*P =* 0.0001) mice and mice that passively received CIS43 (*P =* 0.0159) had a significant reduction (~2 logs) of the liver-stage parasite burden compared with VRC01-AAV–administered or untreated mice when assessed 2 days after challenge (Figure 3B). 2A10-AAV–treated mice had a smaller reduction (~1 log) in parasite burden (Figure 3B). Remarkably, when these mice were further assessed for blood stage infection 6 days after challenge, 9 of 10 CIS43-AAV (*P =* 0.0005) mice and 4 of 5 mice that passively received CIS43 (*P =* 0.0102) had no detectable parasitemia, whereas all mice treated with either 2A10-AAV or VRC01-AAV, or untreated mice, had high levels of parasitemia (Figure 3B). Eight weeks after AAV administration, serum concentrations of total human IgG were significantly higher for 2A10 compared with the CIS43 (*P =* 0.0248) or VRC01 (*P =* 0.0058) groups (averages, 1114.3, 372.0, and 333.2 μg/mL; ranges, 858.8–1302.5, 157.2–458.3, and 65.1-582.2 μg/mL, respectively; Figure 3C). rPfCSP-specific Ab concentrations were significantly higher for 2A10 than CIS43 (averages, 954.4 versus 354.1 μg/mL; ranges, 608.5–1399.3 μg/mL versus 186.9–470.5 μg/mL; *P =* 0.0409; Figure 3D). Similarly, 2A10 had higher average skin concentrations of total human IgG (162.0 μg/g tissue weight) than CIS43 (61.1 μg/g tissue weight; *P =* 0.0169) or VRC01 (56.0 μg/g tissue weight; *P =* 0.0051); ranges were 54.4–235.8 for 2A10, 38.1–110.5 for CIS43, and 6.1–94.3 μg/g tissue weight for VRC01 (Figure 3E). Skin homogenate samples from CIS43-AAV and 2A10-AAV — but not VRC01-AAV administered mice — bound to rPfCSP (average of 59.0 μg/g tissue weight and range of 40.0–115.0 μg/g tissue weight for CIS43; average of 175.9 μg/g tissue weight and range of 58.0–299.0 μg/g tissue weight for 2A10), but the difference in Ab concentrations was not significant between both groups (Figure 3F). In assessing the kinetics of CIS43 in sera from a separate group of mice following CIS43-AAV administration over time, the levels peaked approximately 2 weeks after administration at a concentration of approximately 300 μg/mL and remained stable up to 16 weeks (Supplemental Figure 3A). CIS43 was also sustained in the skin up to 16 weeks (Supplemental Figure 3B). To show that the Ab expressed in vivo following AAV administration was functional, serum from mice administered 2A10-AAV and CIS43-AAV showed comparable binding to rPfCSP as recombinant 2A10 or CIS43 antibody by biolayer interferometry (Supplemental Figure 3C). Taken together, these data demonstrate that CIS43 expressed in vivo by AAV is highly protective and more potent than 2A10 (17), despite 2A10 having higher serum antibody titers.

### CIS43-AAV mediates long-term protection following malaria challenge.

**Figure 4:**
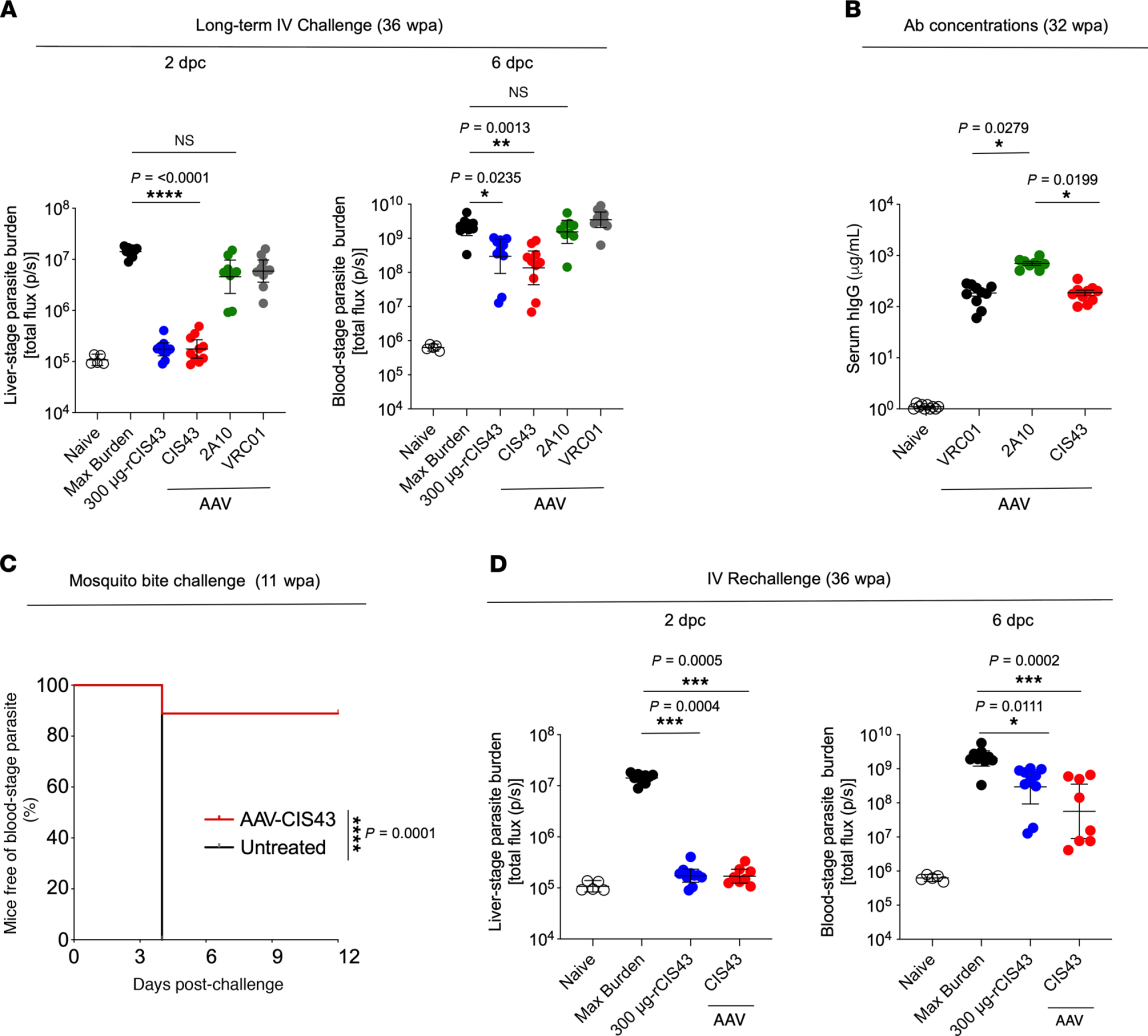
Long-term expression and protective capacity of AAV-induced mAbs. (**A**) C57BL/6 albino mice were challenged i.v. with Pb-PfCSP-LUC SPZ 36 weeks after a single administration of AAV encoding CIS43, 2A10, or VRC01. As a control, rCIS43 (300 μg) administered 2 hours prior to challenge. For all groups, *n =* 10; 2A10-AAV, *n =* 8. *P = 0.0235; **P = 0.0013; ****P ≤ 0.0001. (**B**) Serum hIgG levels of AAV-injected mice shown in **A** were measured by ELISA 32 weeks after AAV administration prior rechallenge (36 weeks). *P* values are shown on the panel. Data represent the mean ± SEM. (**C**) AAV-treated C57BL/6 mice previously protected at 3 weeks after CIS43-AAV administration (Figure 3A) were rechallenged by mosquito bites 11 weeks following the AAV administration. Kaplan-Meier curves, analyzed using the log-rank test, show the frequencies of mice free of parasites as determined through Giemsa staining of blood up to 12 days following challenge. Differences between CIS43-AAV–administered mice and untreated mice are shown (****P = 0.0001). Untreated mice, *n =* 7; CIS43-AAV, *n =* 9. (**D**) C57BL/6 albino mice previously protected when challenged 8 weeks after AAV administration (Figure 3B) were rechallenged i.v. with Pb-PfCSP-LUC SPZ at 36 weeks. Liver burden (left) and parasitemia (right) are shown. *P* values are displayed on the panels. CIS43-AAV, *n =* 8; 300 μg-rCIS43, *n =* 10. Differences in parasite liver burden, parasitemia, or serum antibody concentration between groups were determined using the Kruskal-Wallis test for multiple comparisons with Dunn’s correction. Data represent the geometric mean with 95% CI (**A** and **D**).

A potential advantage of using AAV is the ability to sustain antibody expression in vivo, leading to durable protection. To assess the long-term protective efficacy of CIS43-AAV, mice that received CIS43-AAV, 2A10-AAV, or VRC01-AAV were challenged by the i.v. route 36 weeks after AAV administration. Mice that received CIS43-AAV or 300 μg of CIS43 given at the time of challenge showed a significant reduction of both liver-stage parasite burden (~2 logs, *P* ≤ 0.0001) and blood-stage infection (*P =* 0.0013 for CIS43-AAV and *P =* 0.0235 for CIS43), respectively, compared with untreated mice (Figure 4A). In contrast, mice that received 2A10-AAV had a modest reduction in liver-stage parasite burden (~0.5–1 log, Figure 4A) and had parasitemia similar to that seen in untreated mice (Figure 4A). Thirty-two weeks after AAV administration, consistent with the findings in Figure 3, serum concentration of total human IgG in these mice was significantly higher for 2A10 (701.8 μg/mL) compared with CIS43 (186.9 μg/mL, *P =* 0.0199) or VRC01 (184.5 μg/mL, *P =* 0.0279); ranges were 500.9–1000.1 μg/mL for 2A10, 99.8–348.4 μg/mL for CIS43, and 60.0–283.2 μg/mL for VRC01 (Figure 4B).

A key feature of Ab-mediated protection would be the ability to protect against repeated infection over prolonged periods of time. Thus, CIS43-AAV–administered mice initially challenged by mosquito bites and protected against malaria infection at week 3 (Figure 3A) were rechallenged by mosquito bites 11 weeks following AAV administration. Eight of 9 mice that received CIS43-AAV had no detectable parasitemia up to 12 days after rechallenge (*P =* 0.0001, log-rank test). In contrast, untreated mice exhibited parasitemia by day 4 (Figure 4C). Moreover, CIS43-AAV–administered mice initially challenged by the i.v. route and protected against malaria infection at week 8 (Figure 3B) were rechallenged by the same route at week 36 following AAV administration. Following rechallenge, mice that received CIS43-AAV (*P =* 0.0005) or 300 μg of CIS43 (*P =* 0.0004) at the time of challenge had a significant reduction of both liver-stage parasite burden (~2.5 logs) and blood-stage infection (*P =* 0.0002 for CIS43-AAV and *P =* 0.0111 for CIS43) (Figure 4D). Because antibodies induced after the primary challenge at week 8 may potentially play a role in the protection observed following rechallenge, we determined the level of mouse antibodies against PfCSP 32 weeks after CIS43-AAV administration, just prior to rechallenge at 36 weeks. Mouse anti-PfCSP antibodies were significantly lower than the AAV-expressed CIS43 (OD = 0.5 versus OD = 3.0) (Supplemental Figure 4). Together, these data demonstrate that CIS43-AAV mediates sustained antibody production in vivo and durable protection against primary and repeated malaria challenge.

## Discussion

Potent neutralizing mAbs represent a promising approach for conferring high-level protection against malaria. While mAbs could be used to prevent malaria infection in travelers, health care workers, and military personnel, the greatest public health impact of this intervention would be for seasonal control or elimination campaigns in Africa. Both of these indications would require mechanisms to improve antibody durability. Here, we provide 2 orthogonal approaches for increasing the durability of mAb CIS43 in vivo. The present study shows that CIS43LS has a longer serum *t_1/2_* (38.7 ± 7.8 days) compared with CIS43 (22.3 ± 2.2 days) in NHP, with a titer of ~10 μg/mL at 15 weeks after infusion. The serum *t_1/2_* of CIS43LS reported here is higher compared with a prior study with the HIV mAb VRC01LS (11.8 days) in NHP (18). Remarkably, the *t_1/2_* of VRC01LS was approximately 71 days in humans (34), suggesting that the durability of CIS43LS in humans may be greater than predicted by NHP. The lack of detection of ADA anti-CIS43LS responses in NHP is consistent with previous reports showing that the FcRn binding site mutations, including the LS mutations, are well tolerated and have limited ADA in humans, even after multiple infusions (34, 35).

Although altering the PK profile of antibodies will favorably impact their utility for various clinical use cases, repeated administration might still be required for elimination campaigns of malaria. An alternative solution to sustain Ab titers for longer periods of time with a single administration is the VIP strategy reported here using AAV. AAV vectors have been used safely in humans for gene therapy (36–40) and VIP (41). The demonstration here that persistent VIP expression of CIS43 confers protection up to 36 weeks post-AAV administration (wpa) against a single or sequential malaria challenges provides clear evidence for the promise of this approach for sustaining high levels of antibody. In terms of the protection against repeated infection in this model, immune responses generated after the initial challenge are potential confounding variables to the protection by rechallenge. However, we observed only very low mouse antibody responses following the primary challenge, which likely had limited contribution to the protection we observed after rechallenge.

For clinical translation, a possible limitation for the VIP approach is whether ADA that would limit durability of the antibody are induced. The data presented here show no indication of NHP ADA, and a recent report demonstrated AAV induction of long-lived SIV antibodies in an NHP over 6 years with limited ADA responses (42).

A notable finding was that AAV can result in differential mAb expression in vivo. While it is unclear why the 2A10-AAV expression was significantly higher than that of CIS43 or VRC01, these data are consistent with a previous study, showing very high antibody levels by AAV-encoding 2A10 (23). Importantly, despite having higher antibody titers, 2A10 had more limited protection than CIS43. These data highlight the primary importance of antibody potency for mediating protection, which will be complemented by increased durability.

In summary, this report provides 2 approaches that could be used individually or potentially together to enhance the durability of CIS43 in vivo. Based on the data reported here, a phase I clinical trial evaluating different doses of CIS43LS was initiated this year to assess safety, PKs, and protection against CHMI. As mAb concentrations required for protection against malaria infection in humans remain unknown, the results of the ongoing clinical trial with CIS43LS will provide important information for the future use of mAbs and potentially AAV as modalities to prevent malaria.

## Methods

### Site-directed mutagenesis and rPfCSP probe generation.

CIS43LS was generated through site-directed mutagenesis (GenScript). For rPfCSP production, the codon-optimized synthetic gene corresponding to the full-length circumsporozoite (CS) protein (NCBI gene ID: PF3D7_0304600) of 3D7, a clone of the NF54 strain of *P. falciparum*, was used (GenScript). The 15-mer linear PfCSP peptides that were overlapped by 11 residues spanning the full length of PfCSP were described previously (17) and were synthesized at GenScript.

### Production of CIS43 and CIS43LS.

DNA constructs encoding the heavy and light chain of CIS43 or CIS43LS were expressed in Expi293 (GenScript) or CHO cells to produce both mAbs following purification using a recombinant protein-A column (GE Healthcare). Both mAbs bound equally well to rPfCSP and protected against malaria challenge in vivo. Control mAb 317 (human antibody specific for the NANP-repeat region of PfCSP) was produced in-house using the published sequence of this antibody (43).

### Generation of anti-idiotypic antibody against CIS43 Fab.

Female Balb/c mice (The Jackson Laboratory), 6–8 weeks old, were immunized with 20 μg of CIS43 Fab in PBS mixed with an equal volume of Ribi (MilliporeSigma) adjuvant in a total volume of 100 μL by intramuscular injection, followed by a booster immunization 4 weeks later with the same dose. Ten days following the boost, serum reactivity against CIS43 Fab was tested by ELISA. A germline-reverted CIS43 mAb was generated by cloning the unmutated heavy and light chain genes of CIS43. Mice that strongly reacted to CIS43 Fab but not to germline-reverted CIS43 Fab were selected for cell fusion. Three days before cell fusion, mice with desired reactivity were boosted with 20 μg of CIS43 Fab in a total volume of 100 μL PBS by i.v. injection via the tail vein. Spleens were excised, and purified splenocytes were fused with myeloma cells Sp2/0 (ATCC) in a 2:1 ratio according to established fusion protocols (44). Ten days after cell fusion, hybridoma supernatants were screened for binding to the CIS43 Fab but not to the germline-reverted CIS43 Fab. Following 3 rounds of screening, positive clones were adapted to serum-free hybridoma culture medium (Invitrogen) to facilitate mAb purification. Ultimately, to increase the yield, the DNA constructs encoding the heavy and light chain of selected hybridomas were cloned into the mouse IgG2a expression vectors (GenScript) and expressed in Expi293 (Thermo Fisher Scientific) cells. Antibodies were purified in-house using a recombinant protein-A column (GE Healthcare). Anti-idiotypic mAb 1-1 (produced in-house) was selected for use in the downstream experiments because of its potent binding to CIS43 Fab (produced in-house) and lack of reactivity to germline-reverted CIS43 Fab.

### Processing of skin punches to quantitate mAb.

Macaque or mouse skin punches were processed as previously described (18). Briefly, skin punches were weighed and ground for 1 minute in 1.5 mL tubes containing 400 mL PBS with EDTA-free protease inhibitor (Roche). After grinding, skin homogenates were centrifuged at 16,128*g* for 15 minutes at 4°C. Supernatants were collected and stored at –80°C until testing. Concentrations of passively infused CIS43 and CIS43LS or CIS43-, 2A10-, VRC01-AAV (Ragon Institute, MIT), induced mAb were quantified by ELISA. The amount of each mAb in the mouse skin biopsy homogenate was normalized to the skin tissue weight.

### ELISA.

Binding of CIS43 and CIS43LS to rPfCSP or PfCSP peptides was carried out as described previously (17). Briefly, serially diluted (0.00006–5.0 μg/mL) CIS43, CIS43LS, or control antibodies including the negative control VRC01 (human anti–HIV-1 IgG1) (45) or mAb 317 (43) were added to MaxiSorp ELISA plates (Thermo Fisher Scientific Nunc) coated with 100 μL of rPfCSP (1 μg/mL) or PfCSP peptides (5 μg/mL) per well for 1 hour at room temperature. After a 1-hour incubation, plates were washed and incubated with 100 μL/well of 0.2 μg/mL peroxidase-labeled goat anti–human IgG antibody (KPL, catalog 04-10-06) before adding the ABTS peroxidase (KPL, catalog 50-62-10). The OD was read at 405 nm following addition of stopping solution (100 μL/well). Amino acid sequences included the following: peptide 21, NPDPNANPNVDPNAN; peptide 29, NANPNANPNANPNAN.

To quantitate CIS43, 2A10, and VRC01 induced by AAV administration in serum or skin, ELISA plates were coated for 1 hour with 100 μL of 1 μg/mL of goat anti–human IgG-Fc antibody (Bethyl, catalog A80-104) for measuring total human IgG or plates coated with 100 μL of 1 μg/mL PfCSP to assess PfCSP-specific mAbs. Serum or skin homogenate samples serially diluted (1:100–1:312,500 for serum or 1:20–1:62,500 for skin homogenate, in 5-fold increments) in blocking solution were added to each plate in duplicates, and the ELISA proceeded as described above. A horseradish peroxidase–conjugated (HRP-conjugated) goat anti–human κ light chain antibody (Bethyl, catalog A80-115P) was used as a secondary antibody. A standard curve was generated using each mAb. Antibody quantitation was made from sample dilutions that fell within the linear range of the standard curve. For each specimen, 6 fivefold serial dilutions starting at 1:100 (serum) or 1:20 (skin homogenate) were run in duplicate.

To assess the murine endogenous PfCSP antibody responses after the first malaria challenge, serum samples collected 32 weeks following AAV administration, prior to the second challenge, were diluted 1:100 in blocking solution and tested against rPfCSP by ELISA as described above; mouse PfCSP antibodies were detected with a peroxidase-labeled goat anti–mouse IgG (Invitrogen, catalog 62-6520).

Anti-CIS43 antibody (ADA) responses were evaluated in preimmune macaque serum samples collected 7–14 days prior to antibody infusion and in sera collected 4, 8, 12, 16, and 20 weeks after CIS43LS or CIS43 infusion. For the competition assay, 1 μg/mL CIS43LS or CIS43 was preincubated with pre- and postinfusion serum diluted 1:100 in blocking solution or, as positive control, with serum spiked with an anti-idiotypic mAb recognizing the CIS43 Fab (0.01–1000 μg/mL). The mixture was then added to PfCSP-coated plates. After 1 hour of incubation, plates were washed and incubated with peroxidase-labeled goat anti–human IgG antibody (KPL, catalog 04-10-06) before detection, as outlined above. For the direct assay, plates were appropriately precoated with CIS43LS or CIS43, and macaque or mouse sera diluted 1:100 in blocking solution were added to the plates. ELISA was performed as described above, and macaque ADA were detected with a peroxidase-labeled anti–monkey IgG (Alpha Diagnostic International, catalog 7054).

### ITC.

ITC was carried out as described previously (17). Briefly, CIS43 and CIS43LS were diluted to a concentration of approximately 100 μM (expressed per antigen-binding fragment site) in PBS (pH 7.4) (MilliporeSigma) before injection in 10 μL aliquots into the calorimetric cell containing approximately 1 μM rPfCSP. All titrations were performed at 25°C. Concentration reactants in each experiment were calculated from the protein absorbance at 280 nm. Heat deriving from individual injections of antibody was determined by the integral of the calorimetric indicator. Heat related to the binding of the antibody to rPfCSP within the cell was determined by deducting the heat of dilution from the heat of reaction. The enthalpy variation (ΔH), the association (*K_A_*) and dissociation (*K_D_* = 1/*K_A_*) constant, and the stoichiometry (N) were determined using a nonlinear regression of the individual heats as a function of the concentrations to fit a model that takes into account the binding to 2 sets of sites with different binding energetics for rPfCSP. The variation in Gibbs energy (ΔG), was determined by the equation ΔG = –RTlnKa, where R is the is the universal gas constant (1.987 cal/[K × mol]) and T is the absolute temperature in kelvins. The entropy contribution to Gibbs energy, –T × ΔS, was computed from the known relation ΔG = ΔH — (T × ΔS).

### Kinetic binding assay using biolayer interferometry.

mAbs were purified on a Superdex200 Hi Load 16/600 column to remove aggregates. BLI analysis was performed on a Octet RED384 system using receptors (Acro Biosystems, FCM-H5286, FCM-C5284, and FCM-M82W5) or rPfCSP and PfCSP peptides loaded on HIS1K or SAX biosensors (ForteBio, 18-5122 and 18-5117) and probed into mAbs in a 2-fold dilution series. Assay conditions were individually optimized for each receptor-ligand pair. Data were fit using a 1:1 model on Octet Data Analysis Software version 12.0.2.3 (Molecular Devices LLC). Serial antibody concentrations used were 20, 10, 5, 2.5, and 1.25 μg/mL for pH 6.0 and 2000, 100, 50, 25, and 12.5 μg/mL for pH 7.4 for binding to the receptors, whereas antibody concentrations used for binding to rPfCSP were 2, 1, 0.5, 0.25, and 0.125 μg/mL.

### Mouse challenge studies with chimeric Pb-PfCSP-LUC SPZ.

Malaria challenges to assess the antibody-protective efficacy were done as previously described (17). Briefly, Anopheles stephensi (Nijmegan) mosquitoes were obtained and reared from a colony maintained at the Laboratory of Malaria and Vector Research (NIAID, NIH). Female mosquitoes were allowed to feed on 6- to 8-week-old female Balb/c mice (The Jackson Laboratory) infected with blood-stage Pb-PfCSP-LUC parasites. Eighteen to 21 days following mosquito infections, mosquito salivary glands were dissected and ground in 400 μL of L-15 medium (MilliporeSigma), and viable SPZs were counted in a Neubauer chamber.

Female C57BL/6 mice (6–8 weeks old) from The Jackson Laboratory were i.v. injected with VRC01 or varying concentrations of CIS43 or CIS43LS diluted in PBS (pH 7.4) for a total volume of 200 μL per mouse and challenged 2 hours later with SPZs i.v. (25).

For the VIP studies, female C57BL/6 albino mice (The Jackson Laboratory) were challenged by the i.v. route 8 or 36 weeks after AAV administration. Protected mice were rechallenged 36 weeks after AAV administration. All i.v. challenges were made by the tail vein with 2000 Pb chimeric Pb-PfCSP-LUC SPZ diluted in L-15 medium (100 μL/mouse). For infection assessment, mice were injected i.p. with 150 μL d-luciferin (30 mg/mL) (PerkinElmer) substrate prior to imaging with the IVIS Spectrum in vivo imaging system (PerkinElmer). Parasite liver load was assessed 40–42 hours after challenge, whereas parasitemia was measured 6 days following challenge. Parasite load was quantified by analyzing a region of interest (ROI) in the upper abdominal region and by determining the total flux or bioluminescent radiance (photons/s). For assessment of liver stage infection, a defined ROI around the liver was assessed for all mice, while for parasitemia IVIS was performed on the whole animal. This more comprehensive analysis of the whole animal can result in a shift in the background signal.

The mosquito bite challenge was done as previously described (17). Briefly, C57BL/6 mice were administered with the AAV constructs or were i.v. injected with 300 μg of CIS43, CIS43LS, or VRC01 as detailed above. Mice were anesthetized with 2% avertin (Alfa Aesar), and 10 minutes later, mice were exposed to 6 infected mosquitoes/mouse for 10 minutes to allow sufficient time for the mosquitoes to bite, after which engorged mosquito abdomens were visually inspected for blood to determine whether the mosquito had bitten. Mouse parasitemia was monitored daily by Giemsa staining of blood smears from day 4 through day 12 after infection. The mosquito bite challenge was done 3 weeks (primary challenge) and 11 weeks (rechallenge) after AAV administration.

### PK studies in rhesus macaques.

Male Indian rhesus macaques (5.6–8.2 kg body weight, 2 macaques per group) from Covance were randomly selected and assigned into groups, and they were passively infused i.v. with 10 mg/kg body weight of CIS43LS or CIS43. Blood samples were collected 2 weeks before mAb infusion and after infusion at 5 minutes; 30 minutes; 6 hours; 1, 2, 5, 7, 11, and 14 days; and weekly thereafter up to 140 days. Similarly, skin punches were collected 2 weeks prior to infusion and after mAb infusion at 30 minutes; 2, 7, 11, and 14 days; and every 2 weeks thereafter up to 140 days. Antibody concentrations were quantified by a PfCSP-based ELISA using the KPL kit. Antibodies were quantitated in 6 fivefold serial dilutions run in duplicate starting at 1:20 (serum) or 1:10 (skin homogenate). Blood PK parameters including *t_1/2_*, clearance, and AUC were determined with a 2-compartment model using the WinNonlin software (Pharsight, ref. 18) to estimate the AUC, clearance, and *t_1/2_*.

### HEp-2 cell staining.

Autoreactivity staining assays were performed on HEp-2 cells (Zeus Scientific) per the manufacturer recommendations. mAbs were diluted to 50 and 25 μg/mL using SAVe diluent (Zeus Scientific). A total of 20 μL of the appropriate dilution was coated onto cells fixed on the slide and incubated for 30 minutes at room temperature in a humidified chamber. Slides were rinsed in 1× PBS, washed twice in 1× PBS in Coplin jars for 3–5 minutes, and then stained with 20 μL of FITC-conjugated secondary antibody for 30 minutes in a humidified chamber. Then, slides were rinsed in 1× PBS, washed twice in 1× PBS in Coplin jars for 3–5 minutes, and mounted with 15 μL of mounting media per well and a cover glass (Thermo Fisher Scientific). Slides were imaged on a Nikon Eclipse E800 microscope at 20× in the RGB mode for 2 seconds using SPOT 5.0 software (SPOT Imaging). VRC01, 4E10, VRC07-523, and VRC07-G54W were used as control mAbs for staining and given a score of 0, 1, 2, and 3, respectively, based on their staining intensity. Test mAbs were assigned scores based on visual comparisons of staining intensity to the control antibodies.

### Cardiolipin ELISA.

mAb binding to cardiolipin was tested by ELISA per the manufacturer’s protocol (Inova Diagnostics). Starting at 100 μg/mL, mAbs were tested in a 3-fold series. Assays were validated using positive and negative controls and standards provided in the Quanta Lite ACA IgG III kit (Inova Diagnostics). OD values were converted to IgG anti-phospholipid (GPL) units by linear regression. 4E10 and VRC01 were used as additional positive and negative controls. Cardiolipin binding was scored as follows: no binding for < 15 GPL, indeterminate for 15–20 GPL, low positive for 20–80 GPL, and high positive for > 80 GPL.

### Construction and production of VIP vectors.

DNA segments corresponding to the heavy and light chain variable regions of VRC01 (45), 2A10 (46), or CIS43 (17) mAbs were synthesized (Integrated DNA Technologies) and cloned into the human IgG1 heavy chain and κ light chain constant region framework in VIP expression vectors as previously described (21). Briefly, the resulting vector consists of the heavy and light chain antibody coding regions separated by a picornavirus F2A self-processing peptide sequence that enables expression of both proteins from a single transcript. Transcription is driven by a CASI promoter, and includes a woodchuck hepatitis virus post transcriptional regulatory element (WPRE) and SV40 polyadenylation signal. The entire transgene is flanked by AAV2 ITR elements in which the antibody coding region is driven by VIP. Vector production, quantitation, and storage were done as previously described (21, 22). Briefly, 293T cells were cotransfected with the AAV backbone vector, helper plasmid pHELP (Applied Viromics), and plasmid pAAV2/8 SEED (University of Pennsylvania Vector Core, Philadelphia, Pennsylvania, USA) at a ratio of 0.25:1:2. Culture supernatants were collected at 36, 48, 72, 96, and 120 hours after transfection and pooled. Virus was precipitated by a 40% poly-ethylene glycol solution. Precipitated virus was banded to equilibrium in a 1.36 g/mL cesium chloride solution in a Beckman 70.1 Ti rotor at 60,000 rpm for 24 hours. Collected fractions exhibiting a refractive index between 1.3755 and 1.3655 were combined and diluted in test formulation buffer 2 (TFB2; 100 mM sodium citrate, 10 mM Tris, pH 8). Virus was concentrated and washed with TFB2 by centrifugation at 500*g* at 4°C for 30 minutes using 100 kDa molecular weight cutoff centrifugal filters (MilliporeSigma). Virus was aliquoted and stored at −80°C. Viral genomes were quantified by quantitative PCR.

### VIP vector administration.

After thawing, each AAV construct was diluted with PBS (pH 7.4) to 1 × 10^11^ GC in a 50 μL volume and injected into mice in a single injection (50 μL) into the cranial thigh muscle. Mice were bled by the tail vein, or skin punches were sampled biweekly following AAV administration to assess the Ab expression.

### Statistics.

All data were plotted and graphed using GraphPad Prism, version 8.4.3, unless otherwise stated. For stoichiometry, errors with 95% CI were analyzed from the fits of the data. To measure CIS43, CIS43LS, 2A10, and VRC01 in macaque or mouse serum and skin, standard curves were fitted with a hyperbolic parameter curve, and concentration values were interpolated. For mAb in macaques, differences in serum concentrations between the CIS43LS and CIS43 groups over days 0–140 were calculated using the no-sphericity 2-way ANOVA. Differences in Ab concentrations between groups and liver parasite burden were determined for significance using the nonparametric 1-way ANOVA Kruskal-Wallis test (with Dunn’s correction for multiple comparisons). Parasitemias for the mosquito bite challenge were analyzed by Kaplan-Meier curves using the log-rank test. A statistically significant difference is indicated by *P* < 0.05.

### Study approval.

All NHP and mouse research studies complied with the IACUC of the NIH VRC (protocols VRC-18-755.1 and VRC-19-0824) and Johns Hopkins University (approved protocol permit no. MO18H419).

## Author contributions

NKK, JRF, and RAS conceived, designed, and supervised the project studies. NKK, LDP, KE, YFG, MA, WS, XC, AP, MD, and AS performed experiments. NKK and WS generated mAb 1-1, the CIS43 anti-idiotypic antibody. ABB and SM produced and provided the AAV constructs. NKK, LDP, KE, YFG, MA, AHI, WS, XC, AP, MD, AS, FZ, JRF, and RAS analyzed results from the studies. NKK wrote the first draft of the manuscript. NKK, JRF, AHI, and RAS wrote the final version of the paper. All authors reviewed, edited, and approved the manuscript.

## Supplementary Material

Supplemental data

## Version 1 12/17/2020

In-Press Preview

## Version 2 02/08/2021

Electronic publication
