# Supplementary material for: Enhancing durability of CIS43 monoclonal antibody by Fc mutation or AAV delivery for malaria prevention
Source: JCI Insight. 2021 Feb 8;6(3):e143958. doi: 10.1172/jci.insight.143958 (PMC7934869; doi:10.1172/jci.insight.143958)
Supplement: Supplemental data [file jciinsight-6-143958-s139.pdf]

Supplemental Figure 1

```
CIS43-Heavy         MGWSCIILFLVATATGVHSQVQLVQSGAEVKKPGASVKVSCKASGYTFTSYAIHWVRQAP 60
CIS43LS-Heavy       MGWSCIILFLVATATGVHSQVQLVQSGAEVKKPGASVKVSCKASGYTFTSYAIHWVRQAP 60
                    ************************************************************

CIS43-Heavy         GQRLEWMGWIKAGNGNTRYSQKFQDRVTITRDTSTTTAYMELSSLRSEDTAVYYCALLTV 120
CIS43LS-Heavy       GQRLEWMGWIKAGNGNTRYSQKFQDRVTITRDTSTTTAYMELSSLRSEDTAVYYCALLTV 120
                    ************************************************************

CIS43-Heavy         LTPDDAFDIWGQGTMVTVSSASTKGPSVFPLAPSSKSTSGGTAALGCLVKDYFPEPVTVS 180
CIS43LS-Heavy       LTPDDAFDIWGQGTMVTVSSASTKGPSVFPLAPSSKSTSGGTAALGCLVKDYFPEPVTVS 180
                    ************************************************************

CIS43-Heavy         WNSGALTSGVHTFPAVLQSSGLYSLSSVVTVPSSSLGTQTYICNVNHKPSNTKVDKKVEP 240
CIS43LS-Heavy       WNSGALTSGVHTFPAVLQSSGLYSLSSVVTVPSSSLGTQTYICNVNHKPSNTKVDKKVEP 240
                    ************************************************************

CIS43-Heavy         KSCDKTHTCPPCPAPELLGGPSVFLFPPKPKDTLMISRTPEVTCVVVDVSHEDPEVKFNW 300
CIS43LS-Heavy       KSCDKTHTCPPCPAPELLGGPSVFLFPPKPKDTLMISRTPEVTCVVVDVSHEDPEVKFNW 300
                    ************************************************************

CIS43-Heavy         YVDGVEVHNAKTKPREEQYNSTYRVVSVLTVLHQDWLNGKEYKCKVSNKALPAPIEKTIS 360
CIS43LS-Heavy       YVDGVEVHNAKTKPREEQYNSTYRVVSVLTVLHQDWLNGKEYKCKVSNKALPAPIEKTIS 360
                    ************************************************************

CIS43-Heavy         KAKGQPREPQVYTLPPSRDELTKNQVSLTCLVKGFYPSDIAVEWESNGQPENNYKTTPPV 420
CIS43LS-Heavy       KAKGQPREPQVYTLPPSRDELTKNQVSLTCLVKGFYPSDIAVEWESNGQPENNYKTTPPV 420
                    ************************************************************

CIS43-Heavy         LDSDGSFFLYSKLTVDKSRWQQGNVFSCSVMHEALHNHYTQKSLSLSPGK    470
CIS43LS-Heavy       LDSDGSFFLYSKLTVDKSRWQQGNVFSCSVLHEALHSHYTQKSLSLSPGK    470
                    ******************************:*****.*************
```

Figure S1. Amino acid sequence alignment resulting from the DNA sequencing of CIS43 and CIS43LS heavy chain.
The LS point mutations are boxed in red.

Supplemental Figure 2

A

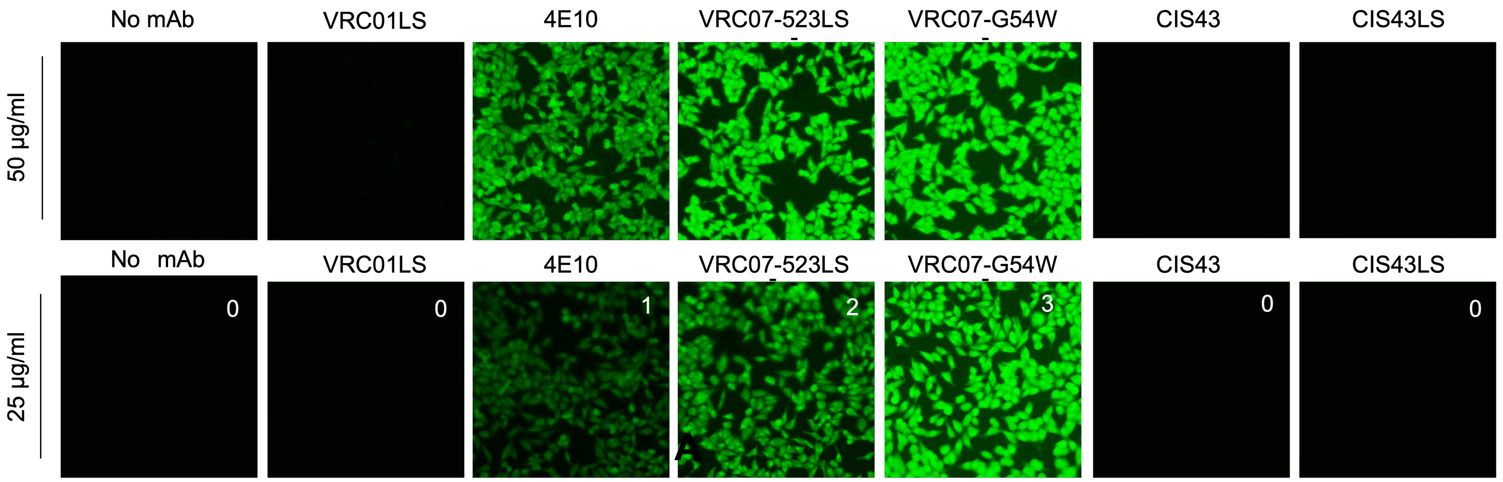

B

mAb	OD at 450 nm		GPL units		Interpretation
	100 µg/ml	33.3 µg/ml	100 µg/ml	33.3 µg/ml	
VRC01LS	0.03	0.03	-0.52	-0.52	Negative
4E10	1.58	1.53	162.34	157.57	High positive
VRC07-523LS	0.24	0.11	20.83	7.02	Negative
VRC07-G54W	0.43	0.18	41.36	14.44	Negative
CIS43	0.04	0.03	-0.46	-0.65	Negative
CIS43LS	0.04	0.04	-0.19	-0.25	Negative

<15	Negative
15-20	Indeterminate
20-80	Low to medium positive
>80	High positive

Figure S2. Autoreactivity of CIS43LS.
(**A**), Representative confocal fluorescence microscopy images of human HEp-2 epithelial cell staining following reactivity with CIS43LS or CIS43. Test antibodies and concentrations (25 and 50 µg) used are indicated. Anti-HIV1 antibodies were used as negative (VRC01LS) and positive (4E10, VRC07-523LS and VRC07-G54W) controls. Images were acquired with 20x objective N.A. = 0.7. Reactivity was assigned scores of 0, 1, 2, and 3 in the 25 µg/mL samples, using the no-mAb sample as baseline.
(**B**) Binding of varying concentrations (100 and 33.3 µg/ml) of mAbs to cardiolipin determined by ELISA. Test and control antibodies are as in (**A**). Values are expressed as optical density at 450 nm ($OD450_{nm}$) and IgG phospholipid signal (GPL) units. One unit represents 1 µg control IgG antibody.

Supplemental Figure 3

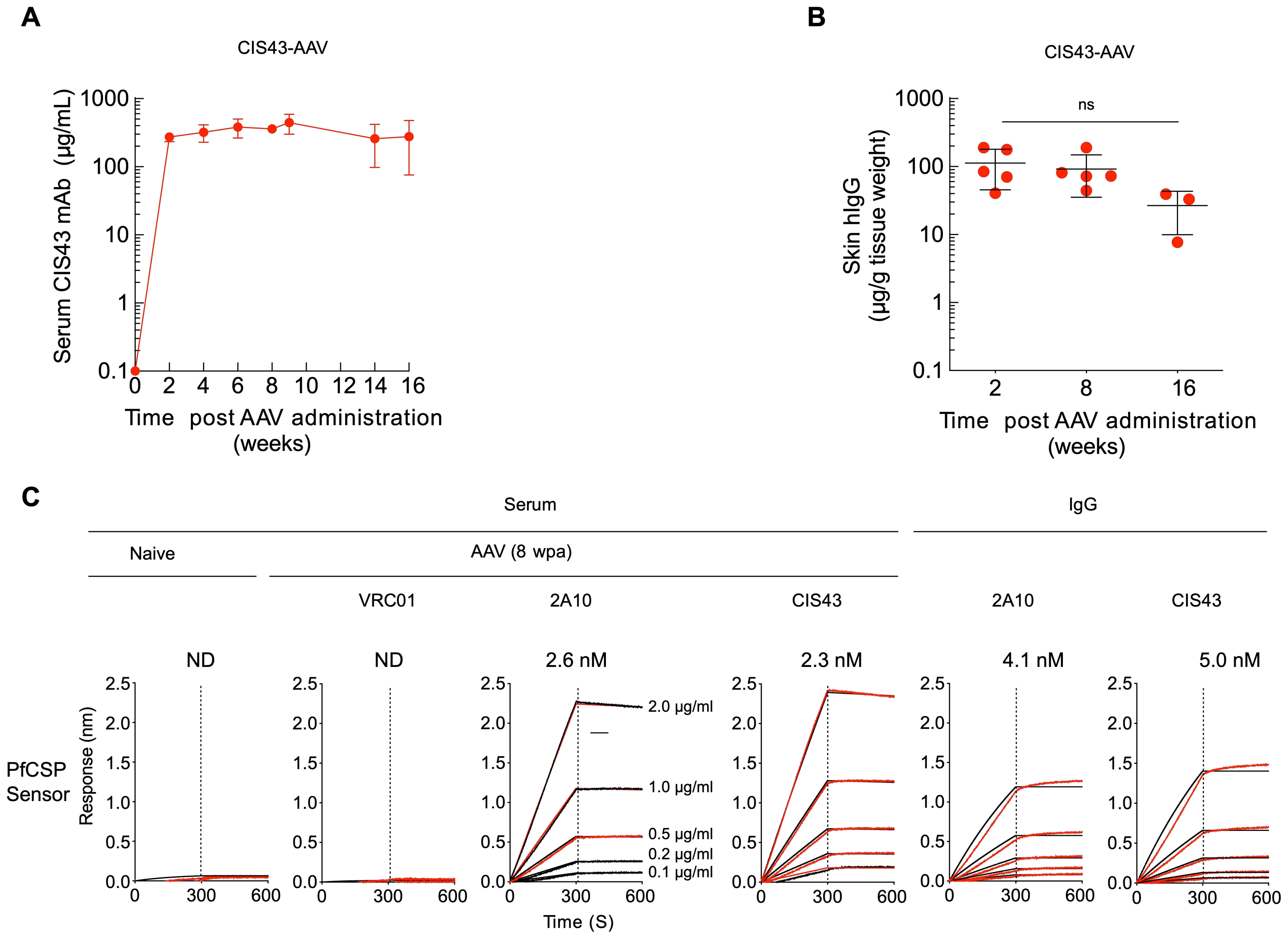

Figure S3. Binding kinetics of CIS43-expressed AAV mAb in mouse serum to PfCSP.
CIS43 mAb levels in serum (**A**) and skin (**B**) at indicated times after IM administration with 10^{11} GC of CIS43-AAV in C57BL/6 albino mice (n = 5). For (**B**), differences in CIS43 concentration at indicated timepoints were determined using the Kruskal-Wallis test for multiple comparisons with Dunn's correction. ns, not significant. Data represent the mean with SD (**A** and **B**). (**C**) Apparent binding affinity of CIS43-, 2A10-, VRC01-AAV in mouse sera to PfCSP, 8 weeks following AAV administration. CIS43 and 2A10 IgG have been used as controls. Antibody binding curves are shown in red (raw data) and black (fitted data). The apparent affinity is displayed as K_D (equilibrium dissociation constant) in nM on each panel. ND, no fit could be determined. mAb serial concentrations (2, 1, 0.5, 0.25, and 0.125 µg/ml) used are displayed.

Supplemental Figure 4

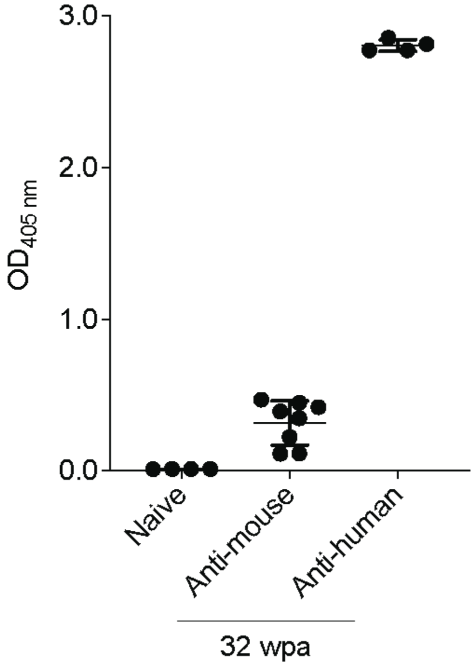

Figure S4. Mouse PfCSP-specific antibody responses in CIS43-AAV-administered mice following the first malaria challenge. Previously challenged and protected mice administered with CIS43-AAV (Figure 3B) were assessed for mouse PfCSP-specific antibodies (anti-mouse) measured by ELISA at 32 weeks just prior to rechallenge. Human IgG induced by CIS43-AAV (anti-human) are shown for comparison. Naïve indicates serum samples collected prior to AAV administration and prior to any challenge. OD_{405nm}, optical density at 405 nm. Data points represent the mean with SEM. Naïve and anti-human, n = 4 per group; anti-mouse, n = 8.

Supplemental Table 1. Summary of binding of CIS43 or CIS43LS to rhesus or human neonatal Fc receptor (FcRn).

Fc Receptor	Sample ID	Kd (nM)	Kd (M)	Kd Error	Kon (1/Ms)	Kon Error	Koff (1/s)	Koff Error	Fold Kd *
Human FcRn pH 6.0	CIS43	54.2	5.4E-08	1.5E-09	4.8E+05	1.2E+04	2.6E-02	3.5E-04	
	CIS43LS	6.2	6.2E-09	6.7E-11	2.7E+05	2.8E+03	1.7E-03	3.6E-06	9.0
Human FcRn pH 7.4	CIS43	ND	No Fits Obtained						
	CIS43LS	234.1	2.3E-07	5.8E-09	4.8E+05	1.1E+04	1.1E-01	8.9E-04	
Rhesus FcRn pH 6.0	CIS43	325.8	3.3E-07	5.4E-09	5.9E+04	9.0E+02	1.9E-02	1.2E-04	
	CIS43LS	25.1	3.1E-08	3.4E-10	3.7E+04	2.2E+02	1.1E-03	1.1E-05	13.0
Rhesus FcRn pH 7.4	CIS43	ND	No Fits Obtained						
	CIS43LS	262.1	2.6E-07	3.8E-09	2.35E+05	2.9E+03	6.15E-02	4.4E-04	

* Fold change in K_D versus unmodified CIS43.
K_D, equilibrium dissociation constant; K_{on}, association rate constant; K_{off}, dissociation rate constant.

Supplemental Table 2. Binding affinities of CIS43 and CIS43LS for human Fc gamma receptors.

**Human Fc Receptor** (pH 7.4)	**Sample ID**	**K_D (nM)**	**K_D (M)**	**K_D Error**	**K_{on} (1/Ms)**	**K_{on} Error**	**K_{off} (1/s)**	**K_{off} Error**	**Fold K_D ***
FcγRI	CIS43	14.84	1.48E-08	2.15E-10	1.87E+05	2.17E+03	2.78E-03	2.41E-05	
	CIS43LS	15.84	1.58E-08	1.96E-10	1.94E+05	1.99E+03	3.08E-03	2.14E-05	1.0
FcγRIIA H167	CIS43	3835	3.84E-06	6.85E-07	1.76E+05	3.11E+04	6.76E-01	1.91E-02	
	CIS43LS	2489	2.49E-06	2.76E-07	2.40E+05	2.60E+04	5.98E-01	1.35E-02	1.5
FcγRIIA R167	CIS43	1479	1.48E-06	1.16E-07	3.48E+05	2.64E+04	5.14E-01	9.79E-03	
	CIS43LS	763.5	7.64E-07	6.45E-08	4.57E+05	3.68E+04	3.49E-01	9.00E-03	1.9
FcγRIIB	CIS43	3132	3.13E-06	8.28E-07	2.59E+05	6.74E+04	8.12E-01	3.81E-02	
	CIS43LS	3803	3.80E-06	1.15E-06	2.30E+05	6.84E+04	8.74E-01	4.11E-02	0.8
FcγRIIIA V176	CIS43	652	6.52E-07	1.55E-08	1.61E+05	3.44E+03	1.05E-01	1.09E-03	
	CIS43LS	550	5.50E-07	2.22E-08	7.87E+04	2.50E+03	4.33E-02	1.08E-03	1.2
FcγRIIIA F176	CIS43	1736	1.74E-06	1.21E-07	1.57E+05	1.06E+04	2.73E-01	5.11E-03	
	CIS43LS	2919	2.92E-06	2.54E-07	4.40+05	3.73E+04	1.29E-01	2.56E-03	0.6
FcγRIIIB NA1 Protein	CIS43	1027	1.03E-06	1.20E-07	4.30E+05	4.80E+04	4.42E-01	1.56E-02	
	CIS43LS	1187	1.19E-06	7.75E-08	3.00E+05	1.88E+04	3.56E-01	6.68E-03	0.9
FcγRIIIB NA2 Protein	CIS43	1283	1.28E-06	1.01E-07	3.27E+05	2.46E+04	4.19E-01	9.08E-03	
	CIS43LS	853.6	8.54E-07	5.20E-08	2.93E+05	1.70E+04	2.50E-01	4.61E-03	1.5

* Fold change in K_D versus unmodified CIS43; K_D, equilibrium dissociation constant; K_{on}, association rate constant; K_{off}, dissociation rate constant. Human polymorphisms: FcγRIIA H167, FcγRIIA H167; FcγRIIA R167; FcγRIIIA V176; FcγRIIIA F176; FcγRIIIB NA1; FcγRIIIB NA2.

Supplemental Table 3: Pharmacokinetic parameters in sera from rhesus macaques administered with CIS43LS or CIS43 (10 mg/kg) by IV route.

Animal ID	mAb	Route	$T_{1/2}$ (Day)	Observed Tmax (Day)	Observed Cmax (µg/ml)	AUC (Day x µg/ mL)	Clearance (mL/Day/Kg)	Serum Concentration (µg/mL)	
								Day 49	Day 98
12M069	CIS43LS	IV	46.5	0.01	261.5	5,467	1.83	37.3	18.8
14D017	CIS43LS	IV	31.0	0.01	313.2	4,107	2.43	28.3	11.5
		AVG	38.7	0.01	287.3	4,787	2.13	32.8	15.1
		SE	7.8	0.0	25.9	680	0.30	4.5	3.7
13C084	CIS43	IV	24.5	0.01	289.2	1,887	5.30	4.1	1.1
15C049	CIS43	IV	20.1	0.01	271.7	1,592	6.28	1.9	0.5
		AVG	22.3	0.01	280.4	1,739	5.79	3.0	0.8
		SE	2.2	0.0	8.8	148	0.49	1.1	0.3

$T_{½}$ (elimination half-life), time taken for the plasma concentration of a drug to fall by half its original value; Tmax, time taken to attain Cmax; Cmax, maximum drug concentration in plasma; AUC (Area Under the Curve), measure of exposure of all the body to the drug; Clearance, measurement of plasma volume from which a drug is entirely removed per day per Kg body weight.
